# A “NOTCH” Deeper into the Epithelial-To-Mesenchymal Transition (EMT) Program in Breast Cancer

**DOI:** 10.3390/genes10120961

**Published:** 2019-11-22

**Authors:** Rohan Kar, Niraj Kumar Jha, Saurabh Kumar Jha, Ankur Sharma, Sunny Dholpuria, Nidhi Asthana, Kundan Chaurasiya, Vivek Kumar Singh, Shuaib Burgee, Parma Nand

**Affiliations:** 1 Indian Institute of Management Ahmedabad (IIMA), Gujarat 380015, India; karrohan1987@gmail.com; 2 Department of Biotechnology, School of Engineering & Technology (SET), Sharda University, Greater Noida 201306, India; saurabh.jha@sharda.ac.in (S.K.J.); chaurasiyakundan1@gmail.com (K.C.); vivekinfo98@gmail.com (V.K.S.); parma.nand@sharda.ac.in (P.N.); 3 Department of Life Science, School of Basic Science & Research (SBSR), Sharda University, Greater Noida 201306, India; ankur.sharma7@sharda.ac.in (A.S.); shuaibburgee741@gmail.com (S.B.); 4 National Centre of Experimental Mineralogy & Petrology (NCEMP), University of Allahabad, Prayagraj 2l1002, India; asthananidhi27@gmail.com

**Keywords:** Notch, EMT, breast cancer, metastasis, invasion, signaling pathway, hypoxia, cytokine, PI3K/Akt

## Abstract

Notch signaling is a primitive signaling pathway having various roles in the normal origin and development of each multicellular organisms. Therefore, any aberration in the pathway will inevitably lead to deadly outcomes such as cancer. It has now been more than two decades since Notch was acknowledged as an oncogene in mouse mammary tumor virus-infected mice. Since that discovery, activated Notch signaling and consequent up-regulation of tumor-promoting Notch target genes have been observed in human breast cancer. Moreover, consistent over-expression of Notch ligands and receptors has been shown to correlate with poor prognosis in human breast cancer. Notch regulates a number of key processes during breast carcinogenesis, of which, one key phenomenon is epithelial–mesenchymal transition (EMT). EMT is a key process for large-scale cell movement during morphogenesis at the time of embryonic development. Cancer cells aided by transcription factors usurp this developmental program to execute the multi-step process of tumorigenesis and metastasis. In this review, we recapitulate recent progress in breast cancer research that has provided new perceptions into the molecular mechanisms behind Notch-mediated EMT regulation during breast tumorigenesis.

## 1. Introduction

Breast cancer (BC) has been widely studied as the result of tempo-spatial aberrations occurring during normal breast tissue development. Similarities between mammary embryonic development and cell-transformation suggest that the underlying mechanisms behind mammary gland development are mostly the ones that are disturbed during various stages of mammary tumorigenesis. Nevertheless, based on the expression of immunohistochemistry (IHC) markers, such as estrogen receptor (ER), progesterone receptor (PR) and human epidermal growth factor receptor 2 (HER2), BC cancer can be split into four major subtypes; namely, ER+/PR+/HER2−, ER+/PR+/HER2+, ER-/PR-/HER2+ and ER-/PR-/HER2−. Further, based upon the usage of “intrinsic” genes, BC can be split into another four groups; namely, luminal A, luminal B, HER2 positive and basal-like. Interestingly, each of the aforementioned IHC-defined subtypes relate to a basic gene expression profiling (GEP)-defined subgroup. For instance, luminal A and luminal B roughly correspond to ER+/PR+/HER2− and ER+/PR+/HER2+ statuses, respectively; albeit a small percentage of ER+/PR+/HER2− tumors (with Ki67 expression) are classified as luminal B type. The subtype with ER−/PR−/HER2− status (also called triple negative breast cancer or TNBC) is primarily comprised of metaplastic, basal-like breast tumors that are rich in interferons and express low levels of claudin [1]. BC cells acquire metastatic abilities that allow them to relocate to sites far away from the primary loci. And often, this metastatic potential determines tumor grade and drives prognosis in patients. 

Epithelial-mesenchymal “transformation” was first studied by Elizabeth Hay using a model of chick primitive streak formation. Later on, researchers described the process as a morphological conversion happening at specific sites in embryonic epithelia to give rise to distinct migratory cells. Consequently, the term “transformation” was replaced with “transition” and the phenomenon came known as epithelial–mesenchymal transition (EMT). BC cells, alike many other cancer cells, utilize EMT that allows the epithelial cells to shed their markers and acquire mesenchymal features [2,3]. Therefore, EMT can also be defined as the trans-differentiation of static epithelial cells into motile mesenchymal cells. EMT is a reversible process (mesenchymal–epithelial transition (MET) being the opposite of EMT) during which epithelial cells acquire mesenchymal properties and exhibit reduced intercellular adhesion and increased motility. The epithelial cells break through the basal membrane and traverse to distant sites by triggering subtle changes in their cytoskeleton architecture. EMT is an important aspect of mammalian growth and development and is constantly involved in processes such as wound healing, stem cell bearings, tumorigenesis, etc. [2,3].

EMT can been classified into three major types: Type 1 EMT that normally operates during embryonic development; Type 2 EMT that is associated with wound healing, tissue regeneration and organ fibrosis; and Type 3 EMT that takes precedence in epithelial cancer cells and participates in metastasis and cancer progression [4]. EMT is driven by a panel of transcription factors that are known to play acute roles during embryogenesis, and also during de-differentiation of cancer cells. They trigger EMT through the transcriptional control of E-cadherin and include members such as Snail-1/2, ZEB-1/2, Twist-1/2, FOXC-1/2, TCF-3 and GSC. Most importantly, Snail and Twist are capable alone, if activated, to prompt a mesenchymal/cancer stem cell-like (CSC) phenotype in human immortalized human mammary epithelial cells [5]. Besides, Twist-1, FOXC-2, Snail-1, ZEB-2 and Twist-2 are reportedly over-expressed in stem-like cells isolated from primary breast carcinomas, in contrast with more differentiated cancer cells [5]. Metastatic breast cancer is largely incurable; therefore, proper understanding of EMT regulation of the metastatic cascade can possibly lead to the development of novel targeted therapeutic strategies.

The Notch signaling pathway plays an important role during normal breast development and during breast cancer development and progression. Different subtypes of mammalian Notch are sensitive to delicate changes in protein levels, and a number of studies have established a key relationship between breast carcinogenesis and Notch deregulation [6]. However, a lot remains to be deciphered especially in the context of breast cancer. Most importantly, Notch cooperates with a panel of signaling pathways, transcription factors, RNAs and a multitude of modulators (activators/inhibitors), thereby making the interactions hard to comprehend [6,7]. For instance, Notch receptors (Notch-1/2/3/4) interact with the epidermal growth factor receptor tyrosine kinase family (RTK) of proteins, such as HER-1, 2, 3 and 4. Over-expressions of HER and/or Notch activity have been reported in BC, thereby identifying Notch as a potent oncogene capable of advancing malignant state in BC [8]. Moreover, Notch maintains HER-induced downstream signals radiated to pathways such as mitogen activated protein kinase (MAPK) and phosphatidylinositol 3-kinase (PI3K). Altogether, these equip the BCs to resist molecular targeted therapies, undergo the process of EMT, and facilitate cellular invasion and metastasis [9]. This review focuses on the EMT program and identifies key Notch-based interactions that could possibly have an important role in driving EMT during BC tumorigenesis. Additionally, the review highlights Notch based interactions in several other cancers, which may act as drivers of the EMT program.

## 2. Epithelial–Mesenchymal Transition

Epithelial–mesenchymal transition (EMT), as introduced earlier, is a key embryonic process that is made possible by the concerted activity of a range of EMT associated transcription factors (EMT-TFs), which are deregulated during tumorigenesis. As a result, the discreet role of EMT-TFs during metastasis and cancer progression remains a subject of intense scrutiny amongst researchers [10]. EMT is a naturally occurring trans-differentiation process that moderates transformation of cell states along the epithelial versus mesenchymal axis and imparts epithelial–mesenchymal plasticity upon epithelial cells. Activation of the EMT program places neoplastic epithelial cells into states where they are almost ready to enter the stem cell compartments [11]. In the early stages of human development, EMT facilitates stem-cell plasticity and morphogenesis and assists gastrulation and organ development. In adults, EMT (and the reverse process, MET, as mentioned earlier) regulates tissue conservation, drives tissue reconstruction and restores cellular homeostasis post inflammatory insults. The EMT process under normal physiological conditions is the same as under pathological conditions because under both these conditions the process is driven by similar regulators, signaling pathways and effectors [12]. In human body, a panel of molecular sub-processes operates in a concerted manner to trigger and facilitate EMT. Some of these include but not limited to the activation of transcription factors, expression of specific cell-surface proteins, reorganization of cytoskeletal proteins, synthesis of ECM-degrading enzymes and changes in the expression pattern of specific microRNAs. EMT is driven by several proteins and factors, such as Snail, Slug, cadherins, Twist, KLF-4, NF-I, TBX-2, SIX, b-Myb, cyclooxygenase-2 (COX-2), ARF-6, FOXA-2, GATA-3, SMAR-1, zinc-finger E-box-binding (ZEB) and basic helix–loop–helix transcription factors (bHLH) [13]. Among all these factors, cadherins play a key role during EMT. Cadherins are molecules located at the adherens’ junctions and are responsible for maintaining proper cell–cell adhesion. There are several types of cadherins, such as E, N, P, VE, proto, desmosomal and FAT cadherins. Notably, E-cadherin (CDH1) and N-cadherin (CDH2) are the ones that facilitate the transition from epithelial state to mesenchymal state that further help the abnormal cells to invade and metastasize nearby and distant tissues. EMT has rarely also been defined as “cadherin switching”; that implies the down-regulation of epithelial proteins E-cadherin and cytokeratins and the up-regulation of mesenchymal proteins, such as N-cadherin, vimentin and fibronectin [2,3,13,14].

EMT is widely regarded as a key driver of tumor malignancy, primarily through the acquisition of malignant phenotypes by epithelial cancer cells. Several studies have reported that cancer cells are capable of acquiring a mesenchymal phenotype and expressing mesenchymal markers, such as α-SMA, FSP-1, vimentin and desmin. Actually, mesenchymal cells are the ones that are localized in the invasive face of primary tumors and are known to enter into the invasion-metastasis cascade [11]. EMT facilitates metastasis, by promoting changes in cell polarity from apical–basal to antero–posterior, by triggering loss of adheren junctions, thereby resulting in a transition from the epithelial to the mesenchymal state, and facilitating the mobility and invasiveness of the metastasizing cancer cells. Epithelial cells are capable of establishing strong cell–cell adhesion with desmosomes, adherens and tight and gap junctions through the activity of proteins such as matrix metalloproteinases (MMPs), E-cadherin and cytokeratin. Incidentally, MMP activity is highly upregulated during EMT, as these enzymes are primarily responsible for extracellular matrix (ECM) degradation and increasing the mobility of mesenchymal-like cells, and hence, promote invasion and metastasis [12]. EMT-derived mesenchymal cells go on to establish secondary tumors at distant sites. Secondary tumors histopathologically resemble the primary tumor from which the cells were initially derived. Further, secondary tumor cells share none of the mesenchymal attributes, which can be usually associated with metastasizing carcinoma cells. This is mainly because, during the formation of secondary tumors, the metastasizing cancer cells shed their mesenchymal attributes by executing the reverse process called MET. The tendency of mesenchymal cells at secondary tumor sites to undergo MET partly reflects the microenvironment (possibly the absence of heterotypic EMT signals) that these cells encounter post-extravasation into the organ parenchyma. Some of the driving factors behind MET are Pax2, bone morphogenetic protein 7 (Bmp7) and Wilms’ tumor 1 (WT1) [11,12,14].

## 3. The Notch Pathway

**Figure 1:**
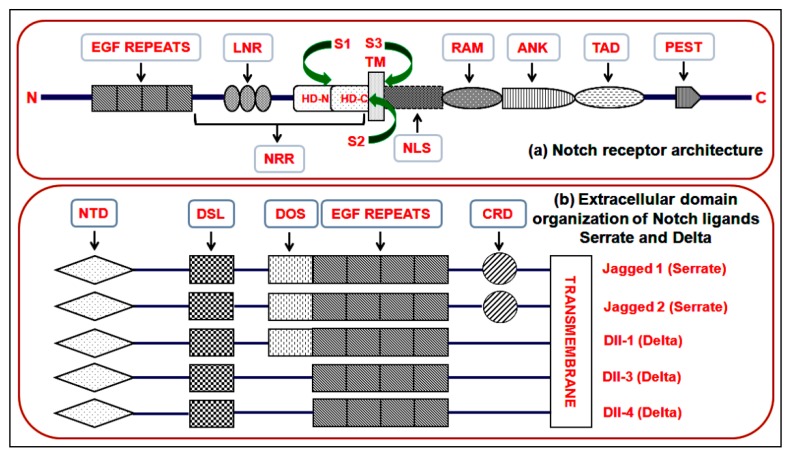
**(a) Notch receptor architecture:** The N-terminal of the Notch receptor contains EGF repeats (approximately 29–36) followed by a Lin-12 Notch repeats (LNR) region. LNR is followed by the heterodimerized region (HD-N and HD-C) that, along with the LNR, forms the negative regulatory region (NRR). The HD region is also the site for the first cleavage (S1) that separates the HD-N and HD-C components. Together, the EGF repeats and NRR form the Notch extracellular domain (NECD). NECD is followed by the transmembrane region (TM), which is the site for the second (S2) cleavage mediated by matrix metalloproteinases (MMPs) (ADAM10/ADAM17). The TM region is followed by the most crucial part of the receptor—Notch intracellular domain (NICD). This region is made up of the nuclear localization sequence (NLS), RBPJ-associated molecule (RAM) domain, six to seven ankyrin (ANK) repeats, transcription activation domain (TAD) and PEST (Pro–Glu–Ser–Thr) domain. S3 is the site for γ-secretase-mediated cleavage that generates the NICD for nuclear transport. **(b) Extracellular domain organization of Notch ligands Serrate and Delta:** The N-terminal domain (NTD) is followed by the Delta–Serrate–LAG-2 (DSL) protein domain and Delta and OSM-11 DOS domain. DOS domains are not present in Dll-3 and Dll-4. Following DOS (Delta and OSM-11-like proteins), domains have varying numbers of EGF repeats. The Serrate ligands additionally have a cysteine-rich domain (CRD) that distinguishes serrate ligands from their Delta counterparts.

**Figure 2:**
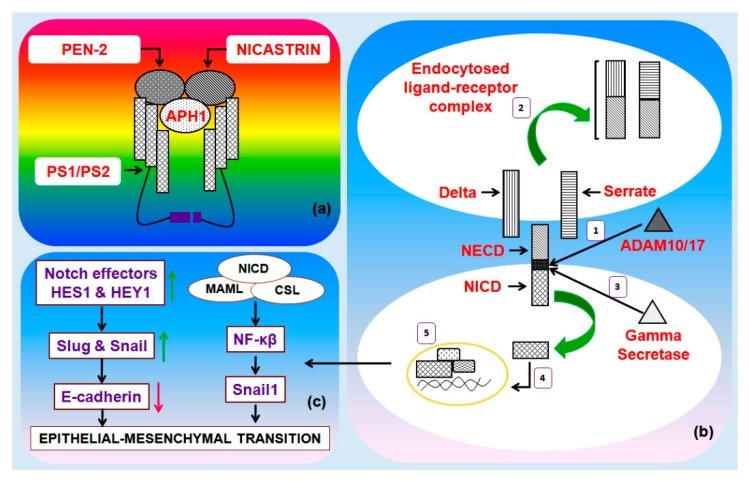
**(a) The gamma secretase (γ-secretase) complex:** It is comprised of presenilin enhancer (PEN-2), anterior pharynx-defective 1 (APH1) and nicastrin. Presenilins (PS1/PS2) are the catalytic components. **(b) The five step canonical Notch pathway:** (1) ADAM 10/17-mediated cleavage frees the Notch extracellular domain (NECD); (2) the receptor-NECD complex is endocytosed by epsin-mediated endocytosis; this initiates the Notch cascade; (3) γ-secretase mediated cleavage releases the transcriptionally active nuclear bound Notch intracellular domain (NICD); (4) NICD enters into the nucleus; and finally (5), NICD forms a complex with the DNA binding protein RBPj and a member of the mastermind-like (MAML) family of transcriptional co-activators to facilitate the transcription of Notch target genes. **(c) Numerous genes are regulated by the Notch pathway that could play a role in epithelial–mesenchymal transitions (EMTs).** CSL, CBF1, Suppressor of Hairless, Lag-1 transcription factor.

Notch receptor and extracellular domain organization of Notch ligands Serrate and Delta plays an important role in canonical Notch pathway (Figure 1a,b). Canonical Notch signaling is triggered by the interaction of Delta/Serrate/LAG-2 (DSL) ligands Jagged and Delta and cognate Notch receptors on adjacent cells [15]. This interaction results in a proteolytic cleavage of the Notch receptor protein at the S2 cleavage site, mediated by either ADAM10 or ADAM17. After this cleavage, the residual part of the Notch protein undergoes a second crucial proteolytic cleavage mediated by the γ-secretase enzyme complex (Figure 2a) [16]. The latter cleavage releases the Notch intracellular domain (NICD), which is free to translocate to the nucleus. Inside the nucleus, NICD forms a complex with the DNA binding protein recombining binding protein suppressor of hairless (RBPJ) and a member of the mastermind-like (MAML) family of transcriptional co-activators (Figure 2b). This protein complex activates the transcription of downstream Notch target genes, which principally includes the members of HEY and HES family (Figure 2c). Notch signaling activity can also be regulated by the post-translational modification of Notch receptors. Notch receptors are glycosylated by the sequential action of peptide-O-fucosyltransferase-1 (POFUT1) and the Fringe GlcNAc-transferases. These modifications modify the affinity of Notch for Delta and Serrate/Jagged ligands. Furthermore, Notch proteins are targets of phosphorylation by glycogen synthase kinase-3β (GSK-3β) and ubiquitination by the E3 ubiquitin ligase F-Box and WD repeat domain containing 7 (FBXW7). These latter modifications adjust the level of NICD, and thus, control the period of signaling [15,16,17,18,19,20,21,22].

## 4. Notch-Mediated EMT in Breast Cancer

**Figure 3:**
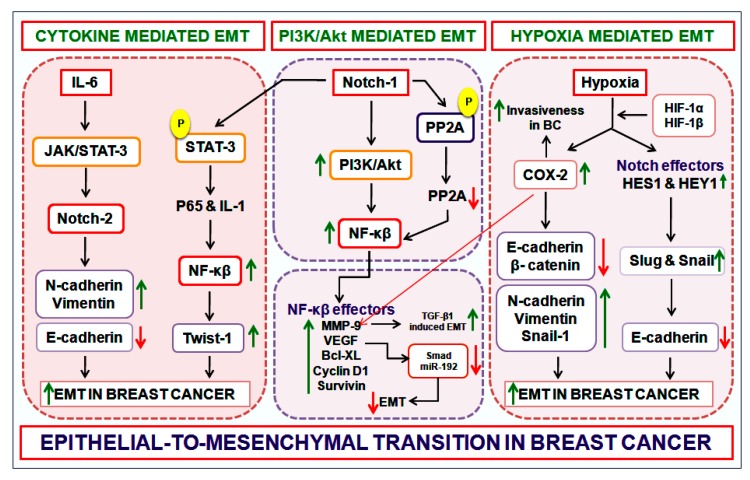
**Notch-mediated epithelial–mesenchymal transition (EMT) cross-talk during breast carcinogenesis:** The EMT process primarily involves the loss of epithelial markers and gain of mesenchymal markers. Once the cells acquire a mesenchymal phenotype, they first intravasate and later extravasate from the blood vessel to establish a distant metastasis. Post-extravasation, there occurs a reverse process called MET (mesenchymal–epithelial transition) that allows the mesenchymal cells to once again revert back to the epithelial type. The above diagram represents the probable cross-talk between three modules that could drive EMT during breast carcinogenesis; viz., the Notch/Cytokine module, the Notch/Akt module and the Notch/Hypoxia module. Inositol blocks both the activity of Notch and Akt and could serve as a potent therapeutic agent targeting EMT. It must be noted that Notch-mediated EMT via Akt and STAT-3 is mediated primarily by NF-κβ. NF-κβ, nuclear factor-kappa β; PP2A, protein phosphatase-2A; IL, interleukin; COX-2, cyclooxygenase-2; BC, breast cancer; VEGF, vascular endothelial growth factor.

EMT is a key phenomenon not only in breast cancer, but across most cancer forms. Therefore undoubtedly, EMT has remained a focus of intensive study in recent times with the sole purpose of identifying critical molecular interactions that might serve as therapeutic nodes and/or improve prognoses in infirmaries [23,24,25]. In the last decade or so, the Notch signal pathway has emerged as a key regulator of EMT during breast carcinogenesis. Despite encouraging initial cues, the precise mechanism of Notch-mediated EMT in breast cancer remains obscure mainly due to the “complexity of unknown interactions.” Notch activation in endothelial cells brings about morphological, phenotypic and functional changes that are consistent with mesenchymal transformation. These alterations include the down-regulation of endothelial markers such as VE-cadherin, Tie1, Tie2, platelet-endothelial cell adhesion molecule-1 and endothelial NO synthase, and over-expression of mesenchymal markers, such as α-SMA, fibronectin and platelet-derived growth factor receptors. In BC patients, elevated Jagged1 expression and hyperactive Notch signaling has been shown to be predictive of poor prognosis. One study suggests that Jagged1-mediated Notch activation triggers the process of EMT via the repression of E-cadherin by Slug [26,27]. The NICD positively regulates Slug expression by stimulating Slug promoter activation. Further, Slug knock-down diminishes the invasion ability of BC cells and reverses the Jagged1/Notch1-induced EMT process signaled by a decrease vimentin expression and increasing E-cadherin levels [28,29,30]. Altogether, these observations prompt us towards the essential role of Notch signaling in modulating EMT, invasion and the growth of BC cells. Herein, we discuss three modules (Figure 3), which present an insight into the process of Notch-mediated EMT in BC. These interactions could be of particular interest, especially bearing in mind the potential therapeutic reputations of these Notch-based processes.

### 4.1. The Notch/Akt Module

Notch regulates Akt activity, and it has been observed that during constitutive Notch-1 activity, the levels of phosphorylated p85 and Akt (Thr308/Ser473) are reportedly very high, thereby signaling PI3K/Akt activation [31,32,33]. The PI3K/Akt pathway plays a significant role during key cellular functions, such as cell growth, proliferation and survival. The NF-κβ transcription factor is a known downstream effector of the PI3K/Akt pathway, and NF-κβ has been reported to be directly involved in both the induction and maintenance of EMT in mammary epithelial cells. One recent study reported that the inhibition of NF-κβ activity in mesenchymal cells can result in the reversal of EMT by abolishing the metastatic potential of mammary epithelial cells [34]. Notch-1 upregulates NF-κβ’s transcriptional activity via the PI3K/Akt cascade, and in doing so, facilitates EMT in human BC cell lines [35]. The activation of this Notch/PI3K/Akt/NF-κβ axis during EMT can be observed by mapping the expression levels of NF-κβ effectors, such as MMP-2/9, VEGF, survivin, Bcl-XL and cyclin D1, which also are Notch downstream targets and have a fundamental role in EMT during carcinogenesis [36,37,38]. For instance, MMP-9 can be associated with TGF-β1-induced EMT. MMP-9 is a downstream effector of TGF-β1 and its inhibition prevents TGF-β1-induced EMT and diminishes cell invasion and metastasis. Moreover, VEGF acts as both activator and suppressor of the EMT program. For example, VEGF promotes tumor angiogenesis and confers efficient tumorigenicity to murine BC cells by facilitating EMT. And conversely, VEGF can also suppress the EMT program by abolishing the expression of Smad3 and miR-192 (a Smad3-dependent microRNA) [39]. Altogether, these observations suggest that Notch-1 signaling can promote the malignant phenotype of BC (EMT, migration and invasion), which may be facilitated in part via the activation of Notch-1/PI3K/Akt/NF-κβ module.

### 4.2. The Notch/Cytokine Module

Cytokines released by the tumor microenvironment can induce EMT in BC. Among these cytokines, interleukin-6 (IL-6) has been reported to promote breast cancer stem cells’ renewal, EMT and metastasis. Most of these phenotypes that have been observed are regulated by the IL-6/JAK/STAT3 cascade, and that primarily involves the IL-6 receptor/GP130 complex. One recent study observed that Notch-2 mediates EMT by interacting with the IL-6/JAK/STAT3 pathway in response to radiation. The study further stated that STAT3-mediated Notch-2 induction results in the downregulation of E-cadherin and upregulation of N-cadherin in radiation treated tumors, a signal indicative of EMT induction. The study presents a crucial interaction by which EMT is regulated by IL-6 in response to fractionated radiation in breast tumors, where Notch-2 upregulation is frequently accompanied a by IL-6-dependent STAT3 activation. Incidentally, high levels of IL-6 in the BC cells are always associated with a more advanced stage of the disease [40]. Another study conducted on Notch-1 over-expressing MCF7 and MCF10A cells showed that the cells acquired features of EMT partly due to the “reverse” activation of STAT3 by Notch-1. Notch-1 triggers the phosphorylation of the STAT3 in BC cells that leads to the activation of p65 and IL-1. The activation of p65 signals in turn signals the activation of the NF-κβ cascade that upregulates *Twist-1* gene expression primarily through nuclear translocation of p65 [41,42,43,44].

Inositol is a known inhibitor of Akt activity, but, there are reports that suggest that inositol can impede EMT by blocking the activity of presenilin-1 (PS1), a key component of the γ-secretase enzyme complex. Downregulation of PS1 impairs the γ-secretase activity that in turn prevents Notch-1 intracellular domain (N1ICD) release. Inositol treated MDA-MB-231 cell lines display high E-cadherin and low MMP-9 levels and decreased activities of effectors such as NF-kβ, COX-2 and Snail-1, which altogether suggest a reversal in the EMT program in these treated cells. Incidentally, the expression of Notch downstream effectors such as HES and HEY is severely downregulated in these treated cells. Further, it has also been observed that β-catenin is redistributed behind the cell membrane, and the cells lose their motility and invasion capacity post-inositol treatment. Similarly, another recent study observed that inositol treatment limits vimentin expression in cells placed behind the wound-healing edge primarily by stabilizing cortical F-actin. Further, lamellipodia and filopodia, the two explicit membrane extensions that enable cell migration and invasiveness, were no longer detectable in MDA-MB-231 cell-lines post-inositol treatment. Besides, the levels of fascin and cofilin, two obligatory required components for F-actin assembly within cell protrusions, were found to be highly reduced post treatment [45,46]. Altogether, these suggest that inositol, due to its ability to impair and Notch-1 activity could be studied further as a blocker of EMT in BC by targeting the Notch/NF-kβ signaling cross-talk.

### 4.3. The Notch/Hypoxia Module

Notch signaling is frequently activated by hypoxia during tumor progression; however, the pathological role of hypoxia-induced Notch signaling in facilitating EMT in BC is poorly understood. Nevertheless, Jagged-2 is considered to be a prognostic marker for the EMT process in BC. At the hypoxic invasive front, both Jagged and Notch have been found to be strongly upregulated in BC, and this activation in BC may trigger EMT and promote cell survival in vitro [47]. Hypoxia elevates the expression of Notch effectors downstream, such as HES1 and HEY1, that in turn upregulate Slug and Snail expression and brings about a reduction in E-cadherin levels in BC cells. Notch-mediated activation of HES1 and HEY1 under hypoxic environment involves factors like HIF-1α and HIF-2α, and it was recently reported that HIF-1α binds to the HES1 promoter directly under hypoxic conditions and that a HIF-1α knock-down lowers both HES1 and HEY1 expression in BC cells [48]. Nuclear HIF-1α-protein expression/translocation may be regulated in part by COX-2. Conversely, hypoxic tumor microenvironment can co-activate COX-2 by utilizing the hepatocyte growth factor (HGF) and TGF-β1 autoregulatory loops, and once activated, COX-2 modulates HIF-1α activation. Research in recent years has revealed that, under hypoxic conditions, aberration in COX-2 expression levels directly links to the EMT process, and to determining the invasive potential of breast tumor. COX-2 over-expression results in down regulation of E-cadherin and β-catenin (epithelial markers), and up-regulation of vimentin, N-cadherin and Snail-1 (mesenchymal markers). Further, COX-2 over-expression also results in increased invasiveness and release of MMP-9 protein. It, however, remains to be seen whether COX-2-mediated EMT is facilitated by the hypoxia/Notch/EMT axis via HIF-1α or COX-2 interacts with HIF-1α independently of Notch [49,50,51,52].

Snail (Snail-1) serves as one of key transmitters of Notch and HIF-1α-mediated EMT signals in BC. Notch modulates Snail-1 expression by two separate but synergistic mechanisms, involving both the direct transcriptional activation of Snail-1 and an indirect phenomenon operating via lysyl oxidase (LOX) that altogether leads to elevated Snail-1 protein levels. This regulation of Snail-1 and LOX expression highlights how Notch and hypoxia signaling mechanisms are integrated and how NICD and HIF-1α collaborate but adopt different roles on the two promoters. Refinement of Notch blockers united with more local containment, e.g., in epithelial tumors at risk of metastasizing, may, therefore, be a fruitful way forward towards improving cancer therapy by targeting hypoxia and Notch-mediated EMT program in BC [49,53]. Nevertheless, these observations altogether hint at the existence of a direct link between hypoxia and Notch in EMT, underscoring the importance of a hypoxic microenvironment in promoting EMT. Further, the role of Notch based interactions during EMT has also been reported in several cancers other than breast cancer, which have been addressed in Table 1.

## 5. Conclusions

In the last decade or so, tumor metastasis has been studied intensely as a key mechanism that drives mortality in BC patients. EMT serves as a mechanism by which cancer epithelial cells acquire mesenchymal characteristics and develop the ability to relocate and establish secondary colonies at distant sites. The mesenchymal tumor cells are highly motile and invasive, and they “metastasize” vigorously. EMT is a well-studied phenomenon, but questions remain in the form of how EMT is driven by signaling pathways in BC and the feedback controls. What are the specific EMT driving proteins and TFs in BC? And how these proteins interact with a multitude of signaling pathways to drive the process as a whole? Nevertheless, the EMT process and interactions are more complex than what they appear *in vitro*. As is the case with this review, research has maintained a consistent stand on the fact that inverse correlations do exist between in vitro migration and E-cadherin levels. However, there still remains a lot to be studied on whether EMT is an absolute necessity for metastasis and invasion. For instance, most breast tumors are invasive, ductal carcinomas and display E-cadherin in primary tumors, thereby refuting the inverse correlation theory [72]. Cancer progression and metastasis is itself a complex and multi-step process, and EMT represents only part of that process. Hence, it becomes perplexing to ascertain whether a particular molecule or pathway under exploration is explicit to the EMT program or is operating in parallel with other programs, such as cell survival and proliferation. To add to this complexity, it is now known that EMT is not only triggered from the program inside tumor cells, but through cues located in the tumor microenvironment, including extracellular matrix, blood vasculature, inflammatory cells and fibroblasts. Owing to the sheer complexity of the process in BC, a lot remains to be deciphered. Recent studies have reported the link between the embryonic signaling pathway, Notch, and EMT in BC, and that has sparked considerable interest in the research community of late. Although the information is limited, the results obtained have provided encouraging molecular evidence justifying the fact that Notch signaling is mechanistically linked with the acquisition of an EMT phenotype in BC cells. In this review, we have presented the collated available data to identify possible Notch-based interactions primarily in BC and later in other cancers as well. Altogether, this will improve our understanding of the Notch based regulatory networks governing EMT during BC metastasis and progression. The involvement of proteins, such as Akt, NF-κβ, STAT3 and HIF-1α, only presents the tip of the iceberg, as it clearly directs our acumen towards the vast array of networks that these proteins govern themselves by, thereby making the EMT phenomenon even more obscure. But there is absolutely no harm in investigating deeper into these interactions with the belief that this shall reveal potential nodes for therapeutic interference, and hence, improve prognosis in patients. Nevertheless, the “complexity of unknown interactions” definitely presents an unyielding challenge and only time shall tell whether targeting Notch or its associated molecular partners directly or indirectly shall prevent or at least limit EMT.

## Figures and Tables

**Table 1:** Several other Notch based interactions during EMT in cancers other than breast cancer.

S. No.	Cancer Type	Associated Factors	Interactions	References
1	Tongue Cancer	JAG1/Notch, LncRNA UCA1/miRNA-124, TGFβ1	UCA1 knockdown increases, whereas miR-124 inhibition decreases TGFβ1-induced EMT and invasion in tongue cancer cells through miR-124 downstream JAG1/Notch signaling	[54]
2	Ovarian Cancer	Notch1, DLK1	Delta-like 1 homolog (DLK1) over-expression promotes ovarian carcinogenesis through Notch activation and EMT induction	[55]
3	Wilms’ Tumor (WT)	JAG1/Notch 1/3, miRNA-539	miR-539 inhibits EMT in WT by inhibiting the expression of JAG1/Notch 1/3 cascade	[56]
4	Oral Squamous Cell Carcinoma (OSCC)	Notch1, EGFR/PI3K/Akt	Notch1 modulates EMT by the activation of EGFR/PI3K/Akt pathway in OSCC	[57]
5	Oral Squamous Cell Carcinoma	Notch1, HNF1A-SA1, STAT3	The transcription factor STAT3 upregulates HNF1A-SA1 and facilitates OSCC progression by activation of the Notch signaling cascade	[58,59]
6	Oral Squamous Cell Carcinoma	Notch1, GLRX3	Glutaredoxin 3 (GLRX3) knock-down limits Notch activity in OSCC by reversing EMT	[60]
7	Hepatocellular Carcinoma	Notch1, Snail-1, N-cadherin, ABCG2, Nanog, Oct4	Notch1-Snail1 signaling pathway contributes to sorafenib resistance by promoting EMT and EMT-mediated CSC features, such as upregulated expression of Snail-1, N-cadherin, ABCG2, Nanog and Oct4, and reduced expression of E-cadherin	[61]
8	Glioblastoma	Notch1/2/3/4, EPN3, WNT/β-catenin	EPN3 may be involved in the Notch and WNT/β-catenin signaling pathways that in turn promotes EMT in glioblastoma cells by activating Slug, Twist and ZEB1, but not Snail-1 or ZEB2	[62]
9	Glioblastoma	Notch1, miRNA-139-5p	miR-139-5p inhibits Notch1 and prevents glioma metastasis and EMT	[63]
10	Gastric Cancer	PS1, miRNA-133a	miR-133a prevents EMT in gastric cells by targeting PS1, a key component in the Notch signaling pathway	[64]
11	Gastric Cancer	Notch1, ZNF-382	KRAB zinc finger protein 382 (ZNF-382) is frequently methylated in gastric cancer and can reverse the EMT program in gastric cancer cells through Notch signaling	[65]
12	Non-Small Cell Lung Cancer (NSCLC)	Notch1, XIST, miR-137	XIST suppresses TGF-β1-induced EMT in NSCLC by regulating the Notch1 pathway	[66]
13	Lung Cancer	Notch1, Numb	Numb functions as a suppressor for a full EMT and thus behaves as a ‘phenotypic stability factor’ by regulating Notch-driven EMT	[67]
14	Pancreatic Cancer	Notch1, HIF-1α	HIF-1α and Notch1 may be involved in regulating the EMT program in MiaPaCa2 cells	[68]
15	Squamous Cell Carcinoma (SCC)	Notch1, Notch3, TGFβ, ZEB1	Notch1 activation and EMT are coupled to trigger SCC tumor initiation in association with TGF-β located in the tumor microenvironment. In response, TGFβ activates ZEB1 that represses Notch3, thereby preventing terminal differentiation	[69]
16	Head and Neck Squamous Cell Carcinoma (HNSCC)	Notch4, HEY1	Notch4 and HEY1 associate to induce cisplatin resistance and promote EMT in HNSCC	[70]
17	Esophageal Squamous Cell Cancer	Notch1, SNHG1, HES1	Small nucleolar RNA host gene 1 (SNHG1) suppresses Notch1 and HES1 and inhibits EMT	[71]
